# Cranberry Proanthocyanidins Neutralize the Effects of *Aggregatibacter actinomycetemcomitans* Leukotoxin

**DOI:** 10.3390/toxins11110662

**Published:** 2019-11-14

**Authors:** Amel Ben Lagha, Amy Howell, Daniel Grenier

**Affiliations:** 1 Groupe de recherche en écologie buccale, Faculté de médecine dentaire, Université Laval, Quebec, QC G1V 0A6, Canada; amelbenlagha@gmail.com; 2 Marucci Center for Blueberry and Cranberry Research, Rutgers University, Chatsworth, NJ 08019, USA; ahowell@njaes.rutgers.edu

**Keywords:** *Aggregatibacter actinomycetemcomitans*, leukotoxin, periodontitis, macrophages, pyroptosis

## Abstract

*Aggregatibacter actinomycetemcomitans* is a Gram-negative bacterium that has been strongly associated with localized aggressive periodontitis. The capacity of *A. actinomycetemcomitans* to produce a leukotoxin (LtxA) that activates pyroptosis in macrophages and induces the release of endogenous danger signals is thought to play a key role in the disease process. The aim of the present study was to investigate the effects of cranberry proanthocyanidins (PACs) on gene expression and cytotoxic activities of LtxA. We showed that cranberry PACs dose-dependently attenuate the expression of genes making up the leukotoxin operon, including *ltxB* and *ltxC*, in the two strains of *A. actinomycetemcomitans* tested. Cranberry PACs (≥62.5 µg/mL) protected macrophages against the cytotoxic effect of purified LtxA. Moreover, cranberry PACs reduced caspase-1 activation in LtxA-treated macrophages and consequently decreased the release of both IL-1β and IL-18, which are known as damage-associated molecular patterns (DAMPs) and contribute to the progression of periodontitis by increasing cell migration and osteoclastogenesis. In addition, cranberry PACs reduced the expression of genes encoding the P2X7 receptor and NALP3 (NACHT, LRR and PYD domains-containing protein 3), which play key roles in pore formation and cell death. Lastly, cranberry PACs blocked the binding of LtxA to macrophages and consequently reduced the LtxA-mediated cytotoxicity. In summary, the present study showed that cranberry PACs reduced LtxA gene expression in *A. actinomycetemcomitans* and neutralized the cytolytic and pro-inflammatory responses of human macrophages treated with LtxA. Given these properties, cranberry PACs may represent promising molecules for prevention and treatment of the aggressive form of periodontitis caused by *A. actinomycetemcomitans*.

## 1. Introduction

Periodontitis, an inflammatory disease initiated by specific bacteria colonizing subgingival sites, affects the tooth-supporting structures, including the connective tissues and the alveolar bone. *Aggregatibacter actinomycetemcomitans* has been strongly associated with the initiation and progression of localized aggressive periodontitis (LAP), which affects specific teeth (incisors and first molars) of young individuals [1,2,3]. In fact, the presence of this Gram-negative bacterium represents a strong risk marker for the initiation of LAP [4,5,6]. Interestingly, it has been reported that in children (6–12 years of age) of parents affected with aggressive periodontitis, the frequencies and quantities of *A. actinomycetemcomitans* is increased compared with children with periodontally healthy parents [7]. *A. actinomycetemcomitans* expresses a number of virulence factors, including a leukotoxin (LtxA), which has been suggested to play a critical role in the pathogenic process of LAP [5,8,9]. LtxA promotes resistance to phagocytosis and affects immune cells by inducing the release of pro-inflammatory cytokines, causing the death of the cells [10]. Although LtxA is secreted in the surrounding environment, it has been also identified in outer membrane vesicles released by *A. actinomycetemcomitans* [11]. These vesicles may contribute to the systemic distribution of the toxin [11] and may modulate the disruption of homeostasis and tissue remodeling processes [12,13]. 

*A. actinomycetemcomitans* LtxA induces the pro-inflammatory cell death or pyroptosis of monocytes and macrophages, which are the most susceptible leucocytes [14]. Pyroptosis, also known as caspase-1-dependent cell death, involves rapid plasma membrane disruption associated with the release of pro-inflammatory intracellular components [15]. This is in marked contrast with apoptosis, which is characterized by the packaging of cellular contents and the non-inflammatory phagocytic uptake of membrane-bound apoptotic bodies [16]. Treating macrophages with LtxA causes the formation of pores in the plasma membrane with a functional diameter of 1.1–2.4 nm; it is a host cell-mediated process that involves caspase-1 activity [10]. The proteolytic enzyme caspase-1 converts the inactive precursors of interleukin-1β (IL-1β) and interleukin-18 (IL-18) into mature inflammatory cytokines [17]. Macrophages exposed to LtxA release high amounts of the pro-inflammatory cytokine IL-1β, which has been shown to mediate bone resorption in a mouse calvarial model [18]. Pathogens have evolved several mechanisms to induce pyroptosis, thus increasing their ability to persist and induce disease. The host and pathogens compete to regulate pyroptosis, and the outcome determines whether the host cells remain viable or die [19].

Over the last decade, bioactive compounds in foods have received considerable attention with respect to oral health [20]. Based on our current knowledge of the etiologic factors and pathogenesis of periodontal diseases, plant polyphenols are a subject of great interest for potential benefits in adjunctive periodontal therapies. The American cranberry (*Vaccinium macrocarpon* Ait) is largely consumed in the form of juice, fresh fruits, dry fruits, and encapsulated powders. Recent studies have provided evidence that cranberry polyphenols, more specifically proanthocyanidins (PACs), possess beneficial properties with respect to oral diseases, including periodontal disease [21,22,23,24]. In a previous study, we showed that cranberry PACs protect oral epithelial cells and macrophages against the toxic effects of certain bacterial components [25]. The aim of the present study was to evaluate the ability of cranberry PACs to (i) down-regulate the expression of LtxA in *A. actinomycetemcomitans*, and (ii) protect macrophages against the deleterious effects associated with pyroptosis induced by LtxA.

## 2. Material and Methods

### 2.1. Cranberry PACs Isolation and Characterization 

Cranberry PACs were extracted from the cranberry fruit (*Vaccinium macrocarpon* Ait.) and purified by solid-phase chromatography, as described previously [26]. The purity and presence of A-type bonds in the PAC preparation were assessed using various analytical methods (^13^C nuclear magnetic resonance, electrospray mass spectrometry, and matrix-assisted laser desorption ionizationtime-of-flight mass spectrometry) [26,27]. The proanthocyanidin molecules consist of epicatechin units with degrees of polymerization (DP) mainly of 4 and 5, containing at least one A-type linkage, as previously reported [26,27]. Purified PACs were dissolved in 50% ethanol at a final concentration of 10 mg/mL and were stored at 4 °C in the dark for up to one month. Preliminary experiments showed that at the dilutions used, the added ethanol had no effects in all assays described below.

### 2.2. Effect of Cranberry PACs on Leukotoxin Gene (ltxA, ltxB, ltxC, and ltxD) Expression

The effect of cranberry PACs on the expression of leukotoxin genes was investigated. The leukotoxin operon includes four genes (*ltxA*, *ltxB*, *ltxC*, and *ltxD*). The *ltxA* gen encodes the toxin structure, *ltxC* encodes the components involved in post-translational acylation, and *ltxB* and *ltxD* encode the components required to transport leukotoxin to the bacterial outer membrane. *A. actinomycetemcomitans* strains JP2 and Y4 were grown to the mid-log phase, which corresponds to an optical density at 660 nm (OD_660)_ of approximately 0.2. Cranberry PACs were then added (30, 40, 50, and 60 µg/mL) and the mixtures were incubated at 37 °C for 6 h under anaerobic conditions. Control cells were incubated in the absence of test compounds. The bacteria were harvested by centrifugation (7000× *g* for 5 min) and were treated with RNA protect bacteria reagent (Qiagen Canada Inc., Montreal, QC, Canada) for stabilization of RNA. Following lysis of bacterial cells, mRNA was isolated and purified using an RNeasy minikit (Qiagen Canada Inc.). The mRNA was reverse-transcribed into cDNA, and a quantitative PCR analysis was performed to determine the levels of *ltxA*, *ltxB*, *ltxC*, and *ltxD* mRNA expression, as described in a previous study [28]. As an internal control for data normalization, the mRNA gene was used. The primers (Life Technologies Inc., Burlington, ON, Canada) used for the quantitative PCR are listed in Table 1. Three independent analyses were carried out in triplicate, and a representative set of data (means ± standard deviations) is presented.

### 2.3. LtxA Purification

*A. actinomycetemcomitans* JP2 [29] was grown (1000 mL) in *A. actinomycetemcomitans* growth medium (AAGM) [30]. LtxA was purified from the bacterial culture supernatant as previously described [31]. 

Moreover, to evaluate the effect of cranberry PACs on LtxA secretion by *A. actinomycetemcomitans* JP2, the purification protocol of LtxA was also carried out using a bacterial culture performed in the presence of 62.5 µg/mL of cranberry PACs. A Pierce^TM^ BCA protein assay kit (Thermo Fisher Scientific, Waltham, MA, USA) was used to quantify proteins in the LtxA preparations. The LtxA preparations were analyzed by sodium dodecyl sulfate (SDS)-12% polyacrylamide gel electrophoresis (PAGE), and Coomassie blue staining. An MTT (3-[4,5-dimethylthiazol-2-yl]-2,5-diphenyltetrazolium bromide) assay (Roche Diagnostics, Mannheim, Germany) was performed to assess the pyroptotic activity of the leukotoxin preparations on macrophage-like cells (see below), following a contact time of 1 or 24 h. 

### 2.4. Cell Culture

U937 human monocytes, purchased from the American Type Culture Collection (Manassas, VA USA; CRL-1593.2), were cultivated in Roswell Park Memorial Institute 1640 medium (RPMI-1640; Life Technologies Inc.) containing 10% heat-inactivated fetal bovine serum (FBS) and 100 µg/mL of penicillin G/streptomycin. Cultures were incubated at 37 °C in a 5% CO_2_ atmosphere. The monocytes (1 × 10^6^ cells/mL) were incubated in RPMI-10% FBS containing 100 ng/mL of phorbol myristic acid (PMA; Sigma-Aldrich Canada, Oakville, ON, Canada) in 6-well microplates for 48 h to allow differentiation into macrophage-like cells [32]. The adherent macrophage-like cells were washed and were incubated in fresh RPMI-1% FBS medium for 24 h prior to stimulation.

### 2.5. Real-Time Cell Viability

To assess the ability of cranberry PACs to impede LtxA-induced cytolysis, macrophages were incubated for 5 h at 37 °C in a 5% CO_2_ atmosphere with purified LtxA (1 µg/mL) in the absence or presence of two-fold serial dilutions of PACs (125 to 3.9 µg/mL; in RPMI containing 1% FBS). Wells with no LtxA or cranberry PACs were used as controls. LtxA-induced cell death was determined using the luminescence RealTime-Glo™ MT Cell Viability Assay (Promega Corporation, Madison, WI, USA) according to the manufacturer’s protocol. Luminescence was quantified using a Synergy 2 microplate reader (BioTek Instruments, Winooski, VT, USA).

### 2.6. Cell Membrane Permeability Assay

The effect of cranberry PACs on plasma membrane integrity of macrophages was assessed using the intracellular dye calcein acetoxymethyl ester (calcein-AM) (Sigma-Aldrich Canada). Briefly, macrophages were differentiated in a black walled, black bottomed, 96-well microplate (Greiner Bio-One North America Inc., Monroe, NC, USA), and were treated with LtxA (1 µg/mL) in the absence or presence of two-fold serial dilutions of cranberry PACs (125 to 7.9 µg/mL in phosphate-buffered saline (PBS; pH 7.2)) in the presence of 5 µL of 1 mM calcein-AM for 3 h at 37 °C in a 5% CO_2_ atmosphere. Calcein-loaded macrophages were observed using an inverted Olympus FSX100 fluorescent microscope (Olympus Canada Inc., Richmond Hill, ON, Canada).

### 2.7. Identification of Apoptotic Cells by Annexin Staining and Determination of Caspase-1 Activation

Adherent macrophages (2 × 10^6^) in 6-well microplates were exposed for 1 h to purified LtxA (1 µg/mL) in the absence or presence of different concentrations of cranberry PACs at 37 °C in a 5% CO_2_ atmosphere. The macrophages were washed twice with ice-cold PBS and were detached by adding Accutase^®^ (1 mL; Innovative Cell Technologies Inc., San Diego, CA, USA) for 7 min (37 °C). The viability of macrophages was determined by staining with annexin V-fluorescein isothiocyanate (FITC) and propidium iodide (PI) (BioLegend, San Diego, CA, USA) for 15 min. To assess caspase-1 activation, the macrophages were stained with AM-VAD-FMK reagent, a fluorochrome inhibitor of caspase-1 (FLICA), according to the manufacturer’s protocol (Thermo Fisher Scientific). Cells were washed to remove unbound reagent. A total of 20,000 cells were analyzed using a Cytomics FC 500 flow cytometer (Beckman Coulter Inc., Indianapolis, IN, USA). Data were analyzed using CXP software (Beckman Coulter Inc., Indianapolis, IN, USA) and Flowing software 2.51 (Perttu Terho, Center for Biotechnology, Turku, Finland) 

### 2.8. Caspase-1 Quantification and Cytokine Analysis

Adherent macrophages were exposed to LtxA (1 µg/mL) in the absence or presence of cranberry PACs at different concentrations, as described above. After treating the cells for 3 h, the supernatants were collected and kept at −80 °C prior to analysis for secreted caspase-1 and cytokines. The adherent macrophages were washed twice with PBS and lysed by adding (100 µL) 0.1% Triton X-100 (in dH_2_O). After incubation for 60 min at room temperature, the cell lysates were collected, subjected to centrifugation, and stored at −80 °C prior to analysis for intracellular caspase-1 and cytokines. The amounts of IL-1β, IL-18, and caspase-1 secreted into the culture medium or contained in the macrophages were quantified by enzyme-linked immunodorbent assay (ELISA) (R&D Systems Inc., Minneapolis, MN, USA) according to the manufacturer’s protocol.

### 2.9. P2X7 and CIAS Gene Expression

Adherent macrophages were exposed to LtxA (1 µg/mL) in the absence and presence of cranberry PACs at different concentrations, as described above. To maintain membrane integrity for the RNA extraction, this experiment was performed in presence of a cytoprotectant (5 mM glycine). Glycine partially inhibits the leukotoxin-induced lysis of macrophages [14]. The presence of glycine has been reported to have no effect on caspase-1 activation or IL-1β secretion by *A. actinomycetemcomitans* LtxA-challenged macrophages [14]. After incubation for 1 h, the cells were collected and their RNA was purified using a RNeasy plus minikit (Qiagen Canada Inc.). The mRNA was reverse-transcribed into cDNA before performing a quantitative real-time PCR analysis to determine the levels of *P2X7* and *CIAS* mRNA expression, as described in a previous study [33]. As an internal control for data normalization, glyceraldehyde-3-phosphate dehydrogenase (*GAPDH*) was used. The primers (Life Technologies Inc.) used for the quantitative real-time PCR are listed in Table 1. Three independent analyses were carried out in triplicate, and a representative set of data (expressed as mean ± SD) is presented.

### 2.10. Determination of Intracellular Reactive Oxygen Species (ROS)

Intracellular ROS generation was measured using a total ROS/superoxide detection kit (Enzo Life Sciences Inc., Farmingdale, NY, USA). Macrophages (10^5^ cells/well) were seeded in 96-well plates and were treated with LtxA (1 µg/mL) at 37 °C for 1 h in the absence or presence of cranberry PACs at different concentrations, as described above. ROS and superoxide generation was then assessed using a commercial kit (ENZ-51010, Enzo Life Sciences Inc.). 

### 2.11. LtxA Binding Assay

FITC-labeled LtxA was prepared using Alexa Fluor^TM^ 488 protein labeling kit (Life Technologies Corporation, Oregon, USA), as described by the manufacturer. Macrophages (2 × 10^6^ cell/well) were exposed to FITC–LtxA (1 µg/mL) in the absence or presence of cranberry PACs at different concentrations in PBS containing 1% BSA and were incubated at 37 °C for 1 h. Cells were washed three times in cell staining buffer (BioLegend) and resuspended in the binding buffer (100 mM HEPES, 140 mM NaCl, 25 mM CaCl_2_, pH 7.4). A total of 20,000 cells were analyzed using a Cytomics FC 500 flow cytometer (Beckman Coulter Inc.). Data were analyzed using the CXP software. Unstained cells and heat-inactivated FITC–LtxA were used as negative controls.

### 2.12. Statistical Analysis

Unless indicated otherwise, all assays were performed in triplicate in two independent experiments. The data are expressed as means ± standard deviations (SD). A one-way analysis of variance (ANOVA) with a post hoc Bonferroni multiple comparison test (GraphPad Software., La Jolla, CA, USA) was used to establish significance of data. All results were considered statistically significant at *p* < 0.01.

## 3. Results

### 3.1. Effect of Cranberry PACs on the Expression of Leukotoxin Genes and Secretion of LtxA 

**Figure 1:**
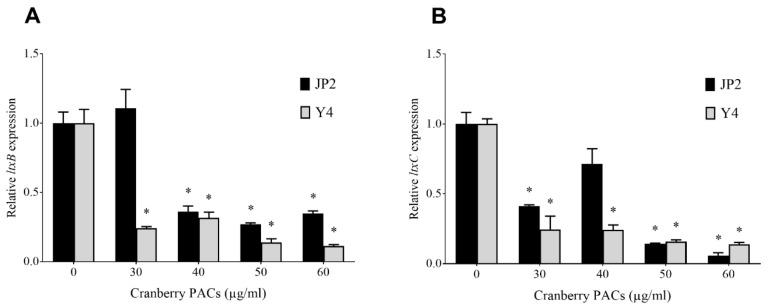
Effects of cranberry PACs on the expression of *ltxB* (**A**) and *ltxC* (**B**) mRNA in two strains of *A. actinomycetemcomitans* (Y4 and JP2). *, significant inhibition at *p* < 0.01.

The effect of cranberry PACs on the expression of leukotoxin genes in two strains of *A. actinomycetemcomitans* (Y4 and JP2) was evaluated by monitoring the expression of *ltxA*, *ltxB*, *ltxC*, and *ltxD* mRNA. At the highest tested concentration (60 µg/mL), cranberry PACs decreased the expression of *ltxB* mRNA (leukotoxin transport to the outer membrane) by 65.3% and 88.7% in the Y4 and JP2 strains, respectively (Figure 1A). Under the same experimental conditions, the cranberry PACs decreased the expression of *ltxC* mRNA (post-translational acylation) by 94.4% and 86.1% in the Y4 and JP2 strains, respectively (Figure 1B). No significant change in the expression of *ltxA* and *ltxD* mRNA was observed.

**Figure 2:**
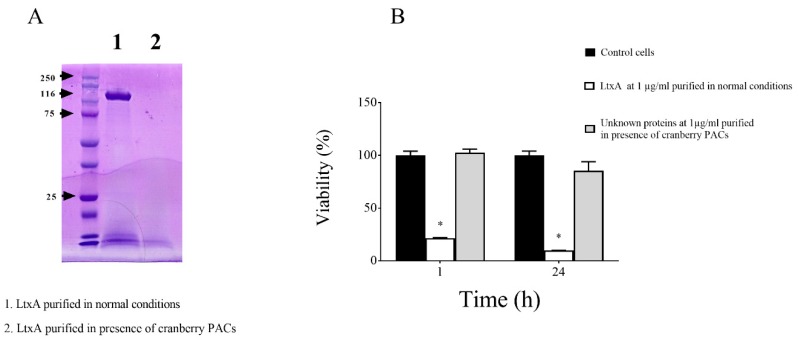
Effects of the presence of cranberry proanthocyanidins (PACs) (62.5 µg/nL) in the culture medium of *A. actinomycetemcomitans* JP2 on the recovery of bioactive LtxA. Sodium dodecyl sulfate-polyacrylamide gel electrophoresis (SDS-PAGE) and Coomassie blue staining (**A**) and cytotoxicity towards macrophages (**B**) of fractions obtained using the LtxA purification protocol. Note: *, significant inhibition at *p* < 0.01.

Thereafter, to investigate the effect of cranberry PACs on LtxA secretion, we compared the amount of purified LtxA recovered from the supernatant of *A. actinomycetemcomitans* JP2 grown in the absence/presence of cranberry PACs (62.5 µg/mL). Growing the bacteria in the presence of cranberry PACs appeared to prevent the secretion of LtxA since the purification protocol did not yield any protein band corresponding to the leukotoxin as determined by SDS-PAGE (Figure 2A). The absence of active LtxA in the preparation recovered from the purification protocol was confirmed by the lack of cytotoxicity towards macrophages (Figure 2B). On the contrary, treating macrophages for 1 h and 24 h with LtxA purified under normal condition resulted in a loss of viability of 78.48% and 90.27%, respectively. 

### 3.2. Effect of Cranberry PACs on Leukotoxin Activity

**Figure 3:**
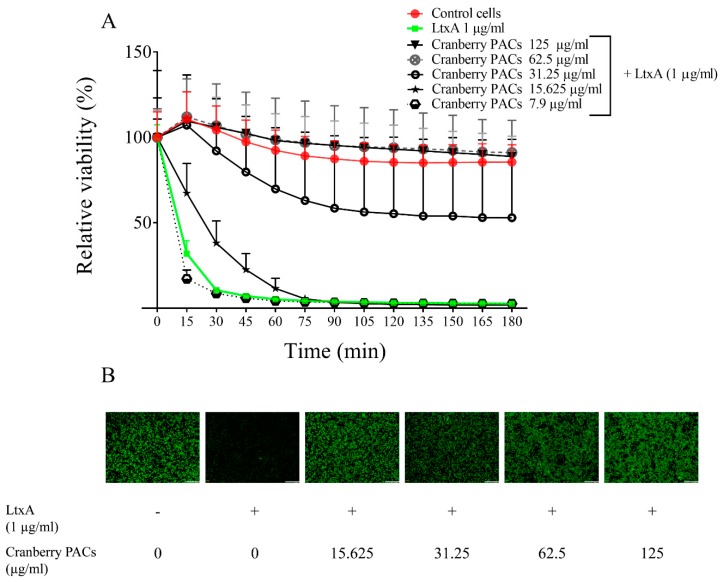
Effect of cranberry PACs on the real-time viability of macrophages exposed to purified LtxA (1 µg/mL). All values are significantly different from those of cells treated with LtxA (*p* < 0.01) (**A**). Immunofluorescence microscopy of macrophages treated (1 h) with purified LtxA in the presence or absence of cranberry PACs; the white bar corresponds to 300 µm (**B**).

The LtxA of *A. actinomycetemcomitans* is considered a key virulence factor because of its cytotoxic effect on macrophages. The capacity of cranberry PACs to protect macrophages against LtxA cytotoxicity was assessed in a time-dependent manner. Macrophage viability decreased by 87.2% following a 1-h exposure to purified LtxA (1 µg/mL), while the cranberry PACs markedly reduced the cytotoxicity of LtxA (Figure 3A). More specifically, 125, 62.5, and 31.25 µg/mL of cranberry PACs reduced the cytotoxicity of LtxA (1-h exposure) by 92.9%, 93.4%, and 64.6%, respectively. To confirm the protective effect of cranberry PACs, a second viability test was performed using calcein-AM, a cell-permeant dye that is converted into a fluorescent molecule by an intracellular esterase. In this assay, pore formation induced by LtxA causes a loss of intracellular fluorescence. The LtxA treatment caused a marked reduction in fluorescence relative to the control cells (Figure 3B). The fluorescence was retained in the cells in the presence of cranberry PACs (≥15.25 µg/mL), suggesting that pore formation did not occur (Figure 3B).

### 3.3. Identification of Apoptotic Cells by Annexin Staining and Determination of Caspase 1 Activation

**Figure 4:**
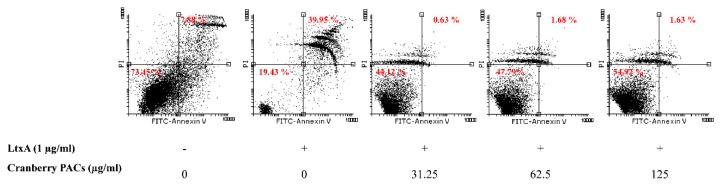
Effect of cranberry PACs on LtxA-induced apoptotic death of macrophages. Cells were stained with annexin V/PI and were analyzed by flow cytometry. The percentage of each cell population is indicated.

**Figure 5:**
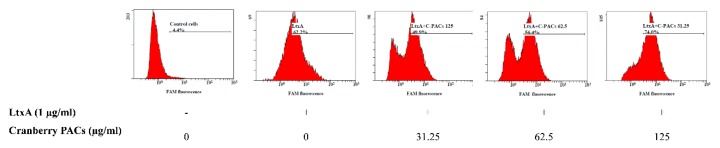
Effect of cranberry PACs on LtxA-induced caspase-1 activation in macrophages. Active caspases were detected using the poly caspase reagent FAM-VAD-FMK FLICA after 60 min in the absence or presence of cranberry PACs.

To better characterize the cell death process induced by LtxA, macrophages were stained with annexin V and PI in the presence or absence of cranberry PACs. Annexin V staining is a marker of early apoptosis, whereas PI stains late apoptotic or necrotic cells. Most cells treated (1 h) with LtxA (1 µg/mL) were annexin V-positive and PI-positive, while cranberry PACs (125 to 31.25 µg/mL) caused a significant reduction in staining (Figure 4). More specifically, 62.5 µg/mL and 125 µg/mL of cranberry PACs reduced annexin V and PI staining by 38.3% and 39.3%, respectively. To confirm that pyroptosis or pro-inflammatory cell death had occurred, caspase-1 activation was assessed by flow cytometry using the fluorescent reagent FAM-VAD-FMK FLICA. A marked shift in the population of caspase-1-positive cells following a treatment with purified LtxA (1 µg/mL) was observed (Figure 5). The dose-dependent reduction in fluorescence following the treatment of macrophages with both LtxA and cranberry PACs indicated that caspase-1 activation is attenuated by cranberry PACS (Figure 5). More specifically, following a 1-h stimulation with LtxA, caspase-1 activity increased by 57.8%, while in the presence of 62.5 μg/mL and 125 μg/mL of cranberry PACs, caspase-1 activation increased by 52% and 4.7%, respectively.

### 3.4. Caspase-1 Quantification and Cytokine Analysis

**Figure 6:**
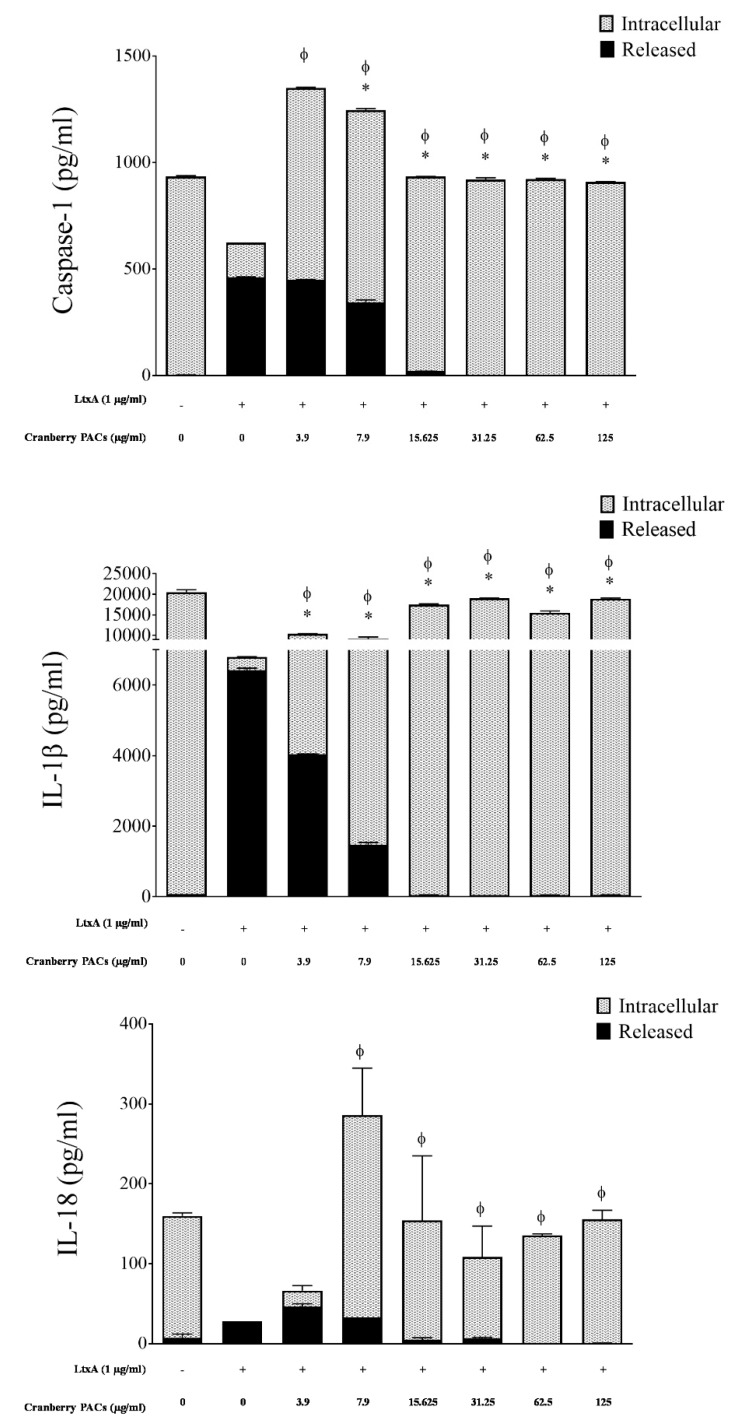
Effect of cranberry PACs on intracellular and released caspase-1 (**A**), IL-1β (**B**), and IL-18 (**C**) from LtxA-treated macrophages. Note: Φ: significant increase in intracellular caspase-1 (*p* < 0.001) relative to control macrophages treated with LtxA alone. *: significant decrease in released caspase-1 (*p* < 0.001) relative to control macrophages treated with LtxA alone.

To confirm that cranberry PACs decreased LtxA-induced caspase-1 activation in macrophages, total caspase-1, IL-1β, and IL-18 (intracellular and released) levels were monitored by ELISA. The levels of caspase-1, IL-1β, and IL-18 released into the culture supernatant of macrophages exposed to purified LtxA (1 µg/mL) increased 117.9-fold, 101.1-fold, and 4.0-fold, respectively, relative to control cells (Figure 6). Conversely, purified LtxA (1 µg/mL) decreased the levels of intracellular pro-caspase-1, pro-IL-1β, and pro-IL-18 767.8-fold, 53.7-fold, and 153-fold, respectively, relative to control cells (Figure 6). In the presence of 125 µg/mL of cranberry PACs, the release of caspase-1, IL-1β, and IL-18 was reduced 100%, 99.3%, and 98.7%, respectively, relative to cells treated with LtxA alone (Figure 6). In contrast, intracellular pro-caspase-1, pro-IL-1β, and pro-IL-18 levels were comparable to those of control cells.

### 3.5. P2X7 and CIAS Gene Expression

**Figure 7:**
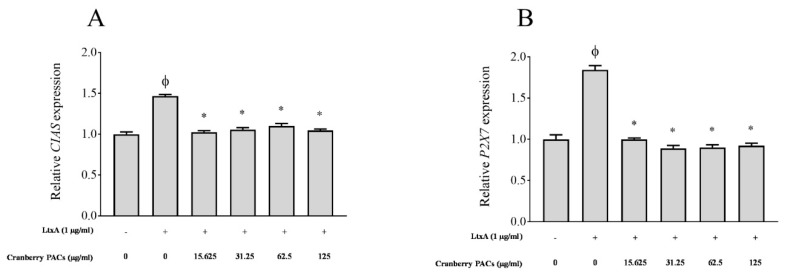
Effects of cranberry PACs on the LtxA-modulated *CIAS* (**A**) and *P2X7* (**B**) expression in macrophages. Note: *, significant inhibition, *p* < 0.01.

The ability of cranberry PACs to modulate the expression of P2X7 and CIAS, which are involved in pyroptosis, was determined. The activation of caspase-1 involves the recruitment of the NAPL3-inflammasome, which is also known as cryopyrin or CIAS. We investigated inflammasome activation by determining the level of CIAS expression. The expression of P2X7 receptors, which are ATP-gated cation channels, was also monitored. The activation of P2X7 receptors and CIAS leads to the rapid formation of membrane pores that are permeable to dyes such as ethidium bromide and to the release of IL-1β and IL-18. Compared to the unchallenged control, LtxA caused a significant 1.5-fold (Figure 7A) and 1.8-fold (Figure 7B) increase in the expression of CIAS and P2X7 mRNA, respectively. In the presence of 125 µg/mL of cranberry PACs, the increase in the expression of CIAS and P2X7 was reduced by 30.2% and 45.8%, respectively (Figure 7).

### 3.6. Measurement of Intracellular ROS and Superoxide

**Figure 8:**
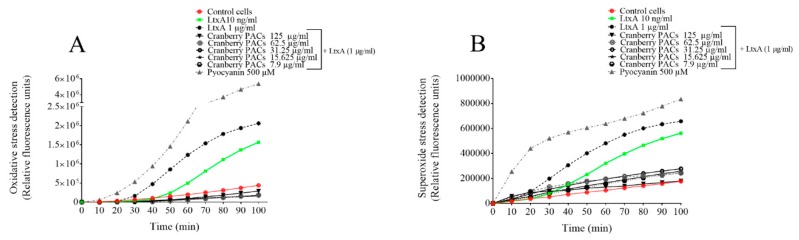
Dose- and time-dependent effects of cranberry PACs on LtxA-induced increases in reactive oxygen species (ROS) (**A**) and superoxide (**B**) production by macrophages.

The activation of the NLRP3 inflammasome has been linked to a number of upstream events, including the production of ROS. The kinetics of the intracellular generation of ROS and superoxide was monitored using a commercial kit. ROS and superoxide production by macrophages increased 6.2-fold and 4.6-fold, respectively, following a 1-h exposure to LtxA (1 µg/mL) relative to control macrophages (Figure 8). ROS and superoxide production was reduced by 92.2% and 72.7%, respectively, in the presence of 125 µg/mL of cranberry PACs, and by 94.7% and 59.5%, respectively, in the presence of 62.5 µg/mL of cranberry PACs (Figure 8).

### 3.7. Binding of FITC–LtxA to Macrophages

**Figure 9:**
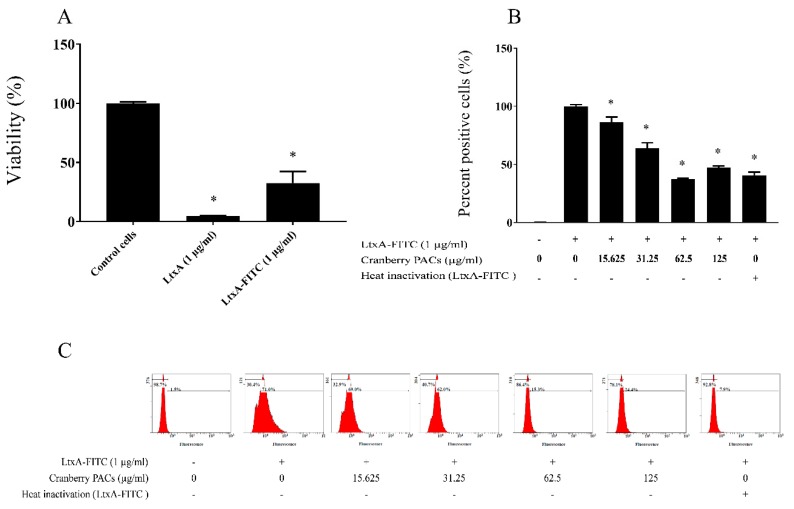
Effects of cranberry PACs on the binding of V-fluorescein isothiocyanate (FITC)–LtxA to macrophages. Cytotoxicity of FITC–LtxA against macrophages as assessed with an MTT test (**A**). Binding of FITC–LtxA to macrophages as determined by measuring the fluorescence using a Synergy 2 microplate reader (**B**) or a flow cytometer (**C**). The percentage of each cell population is indicated. Three independent assays were performed in triplicate.

To better understand the cytoprotective effect of cranberry PACs, we investigated whether the interactions between LtxA and macrophages could be prevented by cranberry PACs. For this analysis, LtxA was labeled with FITC and was found to remain active against macrophages (Figure 9A). As shown in Figure 9B,C, by using flow cytometry and fluorescence measurement, cranberry PACs were found to inhibit the binding of FITC–LtxA to macrophages. More specifically, cranberry PACs at a concentration of 125 µg/mL and 62.5 µg/mL blocked the binding of FITC–LtxA to macrophages by 46.6% and 55.7%, respectively, as determined by flow cytometry.

## 4. Discussion

*A. actinomycetemcomitans* displays considerable genetic heterogeneity, with six distinct serotypes and a large number of variable genes in the pan-genome of this periodontopathogenic bacterium being reported [34,35]. Numerous mutations in the core genome of *A. actinomycetemcomitans* also add to this heterogeneity, which is why the highly virulent JP2 genotype has attracted much attention [6]. The JP2 genotype has a 530-base pair deletion in the promoter of the leukotoxin gene operon [36]. The JP2 genotype is highly leukotoxic and has been strongly associated with the risk of periodontitis compared to non-JP2 genotypes, which have a full-length leukotoxin promoter [4,6]. This leukotoxin allows the JP2 genotype, along with other genotypes in the same environment, to survive or escape from immune cells [37,38]. 

The current treatment for LAP, which often includes mechanical debridement in association with systemic antibiotics, has been associated with a rise in antibiotic resistance in *A. actinomycetemcomitans* [39,40,41,42]. Neutralizing the LtxA activity of *A. actinomycetemcomitans* may represent a promising new strategy for treating LAP. In recent years, evidence has been provided showing that cranberry PACs may have beneficial effects with respect to periodontitis through their action on both periodontopathogens and the host inflammatory response [24]. PACs are catechin oligomers and polymers, and their structure is dependent on both the nature and the types of bonds between the monomers. The aim of the present study was to determine whether cranberry PACs can modulate *ltxA* expression in *A. actinomycetemcomitans* and protect macrophages from the cytotoxic effect of LtxA.

The leukotoxin operon consists of four genes (*ltxA*, *ltxB*, *ltxC*, and *ltxD*). The *ltxA* gene encodes the structure of the toxin, *ltxC* encodes components involved in posttranslational acylation, and *ltxB* and *ltxD* encode components required for the transport of LtxA to the *A. actinomycetemcomitans* outer membrane. The expression of *A. actinomycetemcomitans* LtxA is regulated by environmental factors, such as growth conditions [43]. In the present study, cranberry PACs decreased the expression of *ltxB* and *ltxC* but had no significant effect on the expression of *ltxA* and *ltxD*. Given the involvement of LtxB in exporting LtxA to the outer membrane of the bacteria and the key role played by LtxC in the post-translational modifications to LtxA that are involved in the initial binding of the toxin to host cells [44,45], the ability of cranberry PACs to decrease the expression of these genes may contribute to reducing immune cell death. This is further supported by the fact that growing *A. actinomycetemcomitans* in the presence of cranberry PACs did not allow recovery of bioactive LtxA in the culture supernatant. 

We used a luminescent assay to assess cell metabolic activity and a fluorescent assay to monitor cell membrane disruption and showed that cranberry PACs efficiently protect human macrophages against the cytotoxic effect of LtxA. Early and late apoptotic markers have been previously identified in macrophages exposed to LtxA [46,47,48,49]. In this study, we showed that cranberry PACs reduce the proportion of apoptotic and necrotic macrophages resulting from exposure to LtxA. To the best of our knowledge, the potential beneficial effect of cranberry PACs on neutralizing the cytolysis of macrophages induced by LtxA has not been investigated. Interestingly, Kwamin et al., who investigated the effect on LtxA of seven common plants used as chewing sticks in West Africa, showed that a guava extract efficiently neutralizes *A. actinomycemcomitans* leukotoxicity, whereas the other extracts have no effect [50]. This may be due the presence of PACs in the guava extract, especially in the leaves [51].

IL-1β and IL-18 accumulate as biologically inactive procytokines (pro-IL-1β and pro-IL-18) in the cytoplasm of monocytes and macrophages. The conversion to the biologically active forms requires proteolytic maturation by the cysteine proteinase caspase-1 [52,53], which itself is regulated by an assembly of multiprotein complexes called inflammasomes [54,55]. Kelk et al. reported that the lysis of monocytes and macrophages by LtxA involves the activation of caspase-1 [17]. Therefore, we assessed the effect of cranberry PACs on activation of caspase-1 in macrophages by flow cytometry and showed that cranberry PACs significantly and dose-dependently reduce caspase-1 activation. Given that caspase-1 activation induces the secretion of the biologically active forms of IL-1β and IL-18 [14,17,18], we confirmed that cranberry PACs can indeed decrease caspase-1 activity by showing that they inhibit the release of IL-1β and IL-18 induced by cytolytic concentrations of LtxA. These two inflammatory cytokines are DAMPs and promote the progression of periodontitis by increasing cell migration and osteoclastogenesis [56,57]. NALP3, also known as cryopyrin or CIAS, is a protein complex that stimulates caspase-1 activation and promotes the secretion of proinflammatory cytokines [55]. Previous studies have indicated that the NALP3-inflammasome can be turned on by endogenous danger signals, as well as by pathogen-derived constituents [55,58,59,60]. When macrophages were exposed to LtxA, we observed an increase in the expression of *CIAS-1* relative to cells treated in the presence of cranberry PACs. The fact that cranberry PACs reduced the expression of the *NAPL3*-inflammasone gene may explain how they inhibit cytokine secretion and caspase-1 activation. 

Caspase-1 can be activated in response to various pore-forming toxins and to extracellular ATP [61]. IL-1β secretion is associated with the generation of extracellular ATP, which is a danger signal that activates the purinergic receptor, P2X7R [62]. This causes an K^+^ efflux from cells that in turn activates pro-caspase-1, and thus pro-IL-1β processing [62]. A recent study showed that cell death caused by LtxA is inhibited by oxidized ATP, which is an ATP antagonist, suggesting that P2X7 plays a role in this process [14]. Based on this, we determined the level of P2X7 expression in macrophages exposed to LtxA. We showed that LtxA upregulated P2X7 expression in macrophages, while cranberry PACs prevented this upregulation. ROS are required for purinergic P2X7 receptor-mediated NALP3 inflammasome activation [63,64]. Recent studies showed that ROS generated by NLRP3 activation act as second messengers, whose signaling leads to inflammasome activation [65,66]. We showed in the present study that cranberry PACs reduce the generation of ROS and superoxide by macrophages treated with LtxA. Interestingly, ROS generation is essential for cell signaling, as well as for a number of critical physiological responses. However, the excessive accumulation of ROS can induce cell damage and death. Given this, inhibiting the activation of inflammasomes associated with ROS production may be a promising therapeutic target for treating periodontitis [64].

*A. actinomycetemcomitans* LtxA is known to affect macrophages by binding to the lymphocyte function associated receptor 1 (LFA-1) and to mediate disarrangement of the membrane integrity [10]. To explain the cytoprotective mechanism of action of cranberry PACs, we hypothesized that they could prevent the binding of LtxA to macrophages. We showed that cranberry PACs have the ability to block the interaction between LtxA and macrophages. Interestingly, Krueger et al., demonstrated that a specifically designed and synthesized peptide inhibited LtxA activity by blocking the interaction between LtxA and its LFA-1 receptor [67]. The inhibition of the LtxA–LFA-1 interaction by cranberry PACs may be regarded as an antivirulence strategy to neutralize LtxA cytotoxicity, and they could be used as potent inhibitors of RTX (repeats in toxin) toxins. 

LtxA activity can be neutralized by environmental host-derived proteases and superoxide radicals [43]. To the best of our knowledge, ours is the first report showing that polyphenols can inhibit the gene expression and activity of LtxA. The RTX toxin family, to which *A. actinomycetemcomitans* LtxA belongs, also includes *Escherichia coli* α-hemolysin (HlyA), *Bordetella pertussis* adenylate cyclase (CyaA), *Mannheimia haemolytica* leukotoxin (LktA), and *Actinobacillus pleuropneumoniae* Apx toxin. Studies on the effect of cranberry PACs on other RTX toxins may, thus, be of great interest. 

Epidemiological studies have indicated that the colonization of subgingival sites by *A. actinomycetemcomitans* as the main pathogen and the initiation of inflammation depend on a lack of functional neutrophils in these sites [3,4,7,68,69]. Cranberry PACs, by inhibiting both the gene expression and cytolytic activity of LtxA, may, thus, represent promising candidates for the development of novel therapeutic agents for treating LAP. It would be very interesting to undertake studies on the clinical benefits of incorporating cranberry PACs in oral hygiene products (mouthrinse and chewing gum) or slow periodontal-release devices (inserted in affected periodontal sites).

## Figures and Tables

**Table 1:** Primers used for the quantitative real-time PCR analysis of leukotoxin operon gene expression and inflammasome gene expression in macrophages.

Gene	Primer Sequence
*GAPDH*	Sense: 5′-GGTATCGTGGAAGGACTCATGAC-3′ Antisense: 5′-ATGCCAGTGAGCTTCCCGTTCAGC-3′
*CIAS*	Sense: 5′-CATTAAGATGGAGTTGCTGTTTGAC-3′ Antisense: 5′-CCGACAGTGGATATAGAACAGATAG-3
*P2X7*	Sense: 5′-GAAACGGACTCTGATAAAAGTCTTC-3′ Antisense: 5′-TCTTCCTGTAGTAGTATTCGTTGAC-3′
*16S rRNA*	Sense: 5′-CCTGAATAATGTGGTGATAGTG-3′ Antisense: 5′-CCTCTCTCTATGAACAAGAACG-3′
*ltxA*	Sense: 5′-GTGCTAGGTAAACATCGGTAAAG-3′ Antisense: 5′-GACCACAGAGGCAATTAACC-3′
*ltxB*	Sense: 5′-CTTAGATATCAGTCAGGGAGAAG-3′ Antisense: 5′-CTCTCTGATACTTCGATTAAGCAAC-3′
*ltxC*	Sense: 5′-CATCTCTTGTTTATGACGACTG-3′ Antisense: 5′-GTTTATCGACTTTACCTCCATG-3′
*ltxD*	Sense: 5′-CCAGCAAGTCTCTGAAATTCG-3′ Antisense: 5′-CTTCTTCCGGCACAACTACC-3
